# Bioaccumulation in aquatic systems: methodological approaches, monitoring and assessment

**DOI:** 10.1186/s12302-014-0036-z

**Published:** 2015-01-27

**Authors:** Sabine Schäfer, Georgia Buchmeier, Evelyn Claus, Lars Duester, Peter Heininger, Andrea Körner, Philipp Mayer, Albrecht Paschke, Caren Rauert, Georg Reifferscheid, Heinz Rüdel, Christian Schlechtriem, Christa Schröter-Kermani, Dieter Schudoma, Foppe Smedes, Dieter Steffen, Friederike Vietoris

**Affiliations:** 1 Department of Qualitative Hydrology, Federal Institute of Hydrology, Am Mainzer Tor 1, 56068 Koblenz, Germany; 2 Department of Aquatic Toxicology, Pathology, Bavarian Environment Agency, Demollstr. 31, 82407 Wielenbach, Germany; 3 German Federal Environment Agency, Am Wörlitzer Platz 1, 06844 Dessau, Germany; 4 Department of Environmental Engineering, Technical University of Denmark, DK-2800 Kongens Lyngby, Denmark; 5 Department of Ecological Chemistry, UFZ-Helmholtz Centre for Environmental Research, Permoserstraße 15, 04318 Leipzig, Germany; 6 Department of the Environmental Specimen Bank and Elemental Analysis, Fraunhofer Institute for Molecular Biology and Applied Ecology (IME), Auf dem Aberg 1, 57392 Schmallenberg, Germany; 7 Department of Ecotoxicology, Fraunhofer Institute for Molecular Biology and Applied Ecology (IME), Auf dem Aberg 1, 57392 Schmallenberg, Germany; 8 Deltares, PO Box 85467, , 3508 AL Utrecht, The Netherlands; 9 Masaryk University, Recetox, Kamenice 753/5-A29, 62 500 Brno, Czech Republic; 10 Lower Saxony Water Management, Coastal Defence and Nature Conservation Agency, An der Scharlake 39, 31135 Hildesheim, Germany; 11 Department of Questions of Principle of Water Management, Water Quality of Surface and Ground Water, Water Supply, Ministry for Climate Protection, Environment, Agriculture, Nature Conservation and Consumer Protection of the German State of North Rhine-Westphalia, Schwannstraße 3, 40476 Düsseldorf, Germany

**Keywords:** Ecotoxicology, Environmental quality, Monitoring, Chemical assessment, Environmental quality standards, Water framework directive, Passive sampling

## Abstract

Bioaccumulation, the accumulation of a chemical in an organism relative to its level in the ambient medium, is of major environmental concern. Thus, monitoring chemical concentrations in biota are widely and increasingly used for assessing the chemical status of aquatic ecosystems. In this paper, various scientific and regulatory aspects of bioaccumulation in aquatic systems and the relevant critical issues are discussed. Monitoring chemical concentrations in biota can be used for compliance checking with regulatory directives, for identification of chemical sources or event-related environmental risk assessment. Assessing bioaccumulation in the field is challenging since many factors have to be considered that can affect the accumulation of a chemical in an organism. Passive sampling can complement biota monitoring since samplers with standardised partition properties can be used over a wide temporal and geographical range. Bioaccumulation is also assessed for regulation of chemicals of environmental concern whereby mainly data from laboratory studies on fish bioaccumulation are used. Field data can, however, provide additional important information for regulators. Strategies for bioaccumulation assessment still need to be harmonised for different regulations and groups of chemicals. To create awareness for critical issues and to mutually benefit from technical expertise and scientific findings, communication between risk assessment and monitoring communities needs to be improved. Scientists can support the establishment of new monitoring programs for bioaccumulation, e.g. in the frame of the amended European Environmental Quality Standard Directive.

## Background

Since biota cannot only take up but also accumulate chemicals, monitoring chemicals in aquatic organisms is an essential part of various programs that assess the chemical status of water bodies. Chemicals including hydrophobic organic chemicals (HOCs) and metals are taken up from the aquatic environment either via the water phase or the food which in turn may result in toxicity. In addition, chemicals that tend to partition to sediments can be taken up by direct contact with the sediments. Quantification of chemicals in biota is often analytically challenging. Furthermore, bioaccumulation depends on many abiotic (e.g. site specific water quality like hardness or pH) and biotic (e.g. lipid content, age or sex of an organism) factors that have to be considered in bioaccumulation assessment. As an alternative to measuring chemicals in organisms, the use of passive sampling devices that accumulate chemicals in a reference phase is discussed at the international level. Assessing bioaccumulation is further required for the authorisation of chemicals in many national and international legislative frameworks.

A scientific colloquium, organised by the German Federal Institute of Hydrology and the German Federal Environment Agency in Koblenz (Germany) in 2013, addressed various scientific and regulatory aspects of bioaccumulation in aquatic systems. The colloquium was structured into three sessions in which talks on methodologies, monitoring and assessment of bioaccumulation were presented and discussed with experts from academia, governmental agencies and industries [1]. In this publication, the authors summarise and condense key aspects addressed in the presentations and deliver a brief overview on the outcomes of the event with a focus on the situation in Europe. Since the majority of bioaccumulative compounds belong to the groups of metals and hydrophobic organic chemicals, the authors focus on these chemicals.

### Bioaccumulation as environmental quality criteria

Within the European Water Framework Directive (WFD), Environmental Quality Standards (EQS) for some chemicals in biota (EQS_biota_) have been set, and measures have to be taken by the member states if an EQS is exceeded [2]. The European Commission has strengthened the significance for monitoring bioaccumulation in aquatic organisms by amending the EQS directive in August 2013 [3]: The directive states that ‘very hydrophobic substances accumulate in biota and are hardly detectable in water even using the most advanced analytical techniques. For such substances, EQS should be set for biota‘. As a consequence, in addition to hexachlorobenzene, hexachlorobutadiene and mercury, EQS_biota_ have been set for eight new priority substances or substance groups. These include polybrominated diphenyl ethers, fluoranthene, benzo[a]pyrene, dicofol, perfluorooctanesulfonic acid (PFOS) and its derivatives, dioxins and dioxin-like compounds, hexabromocyclododecane (HBCDD), as well as heptachlor and heptachlorepoxide. EQS for new priority substances have to be taken into account for monitoring programs that have to be submitted by the end of 2018 and have to be complied with by the end of 2027.

In the EQS directive (2013/39/EU), EQS_biota_ values usually refer to concentrations in fish, with the exception of fluoranthene and further polycyclic aromatic hydrocarbons that need to be monitored in crustaceans or molluscs. For dioxins and dioxin-like compounds, the EQS_biota_ refers to fish, crustaceans and molluscs. As long as the equivalent level of protection is complied with, the member states can apply an EQS for an alternative matrix or use another biota taxon [3]. For the majority of priority substances, approximate EQS for the water phase (EQS_water_) can be obtained by dividing EQS_biota_ with bioaccumulation factors (BAFs) [4].

### Derivation of EQS_biota_

EQS_biota_ are derived for the most sensitive subject of protection (e.g. human health, wildlife) and for substances that particularly accumulate in aquatic biota such as fish. The European Technical Guidance Document for Deriving Environmental Quality Standards [4] requires a derivation of quality standards in biota (QS_biota_) for chemicals with a bioconcentration factor (BCF) ≥ 100 or a biomagnification factor (BMF) > 1. BCF is defined as the ratio between the chemical's concentration in organism (*c*_organism_) to the respective concentration in water (*c*_aq_) whereas BMF is the ratio between the chemical's concentration in predator (*c*_predator_) to concentration in prey (*c*_prey_):

1 $$ \mathrm{B}\mathrm{C}\mathrm{F}=\frac{c_{\mathrm{organism}}}{c_{\mathrm{prey}}} $$

2 $$ \mathrm{B}\mathrm{M}\mathrm{F}=\frac{c_{\mathrm{predator}}}{c_{\mathrm{prey}}} $$

Both, BCF and BMF, are usually determined in laboratory tests according to Organization of Economic Co-operation and Development, OECD, test 305. BMF values are not determined by the ratio of chemical concentration in predator and prey but by a kinetic dietary BMF (BMF_k_) (compare the ‘Assessing bioaccumulation potential of chemicals’ section) [5]. If no measured BCF or BMF values are available, other indicators for a high bioaccumulation potential such as a 1-octanol to water partition coefficient (log K_OW_) ≥ 3, biota monitoring data or high toxicity to mammals and birds require biota assessment.

For wildlife, QS_biota_ are derived from the no observed adverse effect level (NOAEL_oral_) that has no effects on the endpoints measured in the test organism after oral uptake in the laboratory. NOAEL_oral_ are multiplied with the ratio of body weight to daily feeding rate to obtain the no observed effect concentration for the food (NOEC_oral_):

3 $$ {\mathrm{NOEC}}_{\mathrm{oral}}={\mathrm{NOAEL}}_{\mathrm{oral}}\frac{\mathrm{body}\kern0.24em \mathrm{weight}}{\mathrm{feeding}\kern0.24em \mathrm{rate}} $$

By dividing the lowest NOEC_oral_ by a safety factor, a QS for secondary poisoning is obtained (QS_biota,secpois_). The method can be improved by using NOAEL_oral_ from laboratory test organisms and data of food uptake as well as body weight of wildlife. In the marine environment, biomagnification potential is further taken into account when deriving QS_biota,secpois_ since there are mammals that feed on other mammals. In contrast to QS for secondary poisoning, QS_biota_ for human health can be obtained by threshold levels of other directives or by a tolerable threshold level for the consumption of fishery products (QS_biota,hhfood_). The latter considers that less than 10% of the tolerable threshold level occurs via fishery products and that 115 g of fish are consumed per person per day. For both protection goals, QS_biota_ are generally in the same range, and the most conservative value has been defined in the EQS directive. For PFOS, for example, 33 and 9.1 μg kg^−1^ wet weights have been derived as QS for secondary poisoning and human health, respectively, and the lower QS has been adopted in the directive.

### Assessing bioaccumulation potential of chemicals

For the registration of chemicals, bioaccumulation potential has to be assessed in aquatic animals. For HOCs, log K_OW_ is used as a screening tool. Indeed, threshold levels differ between regulations. In Europe, the threshold value for industrial chemicals having a production volume of ≥ 100 t/a and for agricultural pesticides is log K_OW_ > 3 [6–8] whereas for veterinary medicines, it is log K_OW_ ≥ 4 [9,10], and for human pharmaceuticals, it is log K_OW_ ≥ 4.5 [11]. In addition to log K_OW_, the BCF value is used for the identification of bioaccumulative substances. According to EU regulation 253/2011 [12], in the context of assessing persistent, bioaccumulative and toxic substances^a^ as well as very persistent and very bioaccumulative substances^b^, substances are bioaccumulative with a BCF > 2,000 and very bioaccumulative with BCF > 5,000. Similarly, the Stockholm Convention regards persistent organic chemicals (POP) with BCF > 5,000 as bioaccumulative [13].

BCF values are experimentally determined by aqueous exposure according to OECD test 305 [5]. The test consists of two phases: the exposure (uptake) and post-exposure (depuration) phases. Fish are usually exposed in a flow through system to at least one concentration of the test substance for 28 days. The duration can be lengthened if necessary or shortened if it is demonstrated that steady-state is reached earlier. If equilibrium concentration between fish and water is achieved within the exposure phase (concentration in fish does not increase further), BCF at steady state (BCF_SS_) is calculated:

4 $$ {\mathrm{BCF}}_{\mathrm{SS}}=\frac{c_{\mathrm{fish},\mathrm{S}\mathrm{S}}}{c_{\mathrm{aq},\mathrm{S}\mathrm{S}}}, $$

with *c*_fish,SS_ and *c*_aq,SS_ being chemical concentrations in fish and water at steady state, respectively.

Following the uptake period, fish are transferred to clean water to measure the depuration of the test substance. And a kinetic BCF (BCF_K_) is obtained by dividing the uptake rate constant (k_1_) with the depuration rate constant (k_2_):

5 $$ {\mathrm{BCF}}_{\mathrm{k}}=\frac{k_1}{k_2} $$

Since growth and lipid content of test organisms can have major impact on bioaccumulation, data should be corrected for both parameters. Care should be taken that the correct lipid extraction procedures are used for the determination of lipid content in fish samples from bioaccumulation studies [14]. For verification of BCF estimates that are based on K_OW_ and quantitative structure activity relationships, a minimised test design can be applied provided that specific criteria are met. This can reduce cost and animal use [5]. In general, the performance of BCF studies with highly hydrophobic chemicals with log K_OW_ > 5 and water solubility below approximately 0.01 to 0.1 mg/L is difficult. If a stable aqueous concentration of the test substances cannot be maintained, a dietary exposure test is required whereby fish are fed daily with pelleted fish food dosed with one or more test substances [5]. For HOCs, the pelleted food is contaminated by mixing with fish or vegetable oil that has been dosed with the test substances. HOCs can also be dissolved in organic solvent and sprayed directly on pelleted food [15]. After feeding test animals with contaminated food for 7 to 14 days, a depuration phase follows that typically lasts for up to 28 days. This approach yields a BMF_k_ and is calculated by multiplying the chemical assimilation efficiency (α) with the feeding rate constant (Ι) and dividing the product by the overall depuration rate constant (k_2_):

6 $$ {\mathrm{BMF}}_{\mathrm{k}}=\frac{a\times \mathrm{I}}{k_2} $$

Since a generally accepted methodology for comparing BMF and BCF values is still missing, independent threshold values for both endpoints are required. Furthermore, experimentally determined BMF values are generally not directly comparable with field BMFs (BMF_field_) which represent a BAF reflecting aqueous and dietary exposure. Laboratory bioaccumulation studies carried out according to OECD [5] have a high use of test animals and consequently we recommend the development of *in vitro* methods for preliminary testing.

For registration of chemicals, bioaccumulation assessment is exemplified for agricultural pesticides: In the EU, pesticides having a log K_OW_ > 3 or other evidence for increased bioconcentration potential and which are regarded as persistent in water (<90% substance loss by hydrolysis in 24 h) have to be tested in bioconcentration studies [6]. If a BCF > 1,000, an elimination < 95% after 14 days and a substance stability in water > 100 days have been measured in the laboratory test, chronic toxicity in fish and biomagnification in aquatic food chains have additionally to be regarded for risk assessment of pesticides. Biomagnification potential of pesticides is taken into account by calculating secondary poisoning of birds and mammals that feed on fish as well as of birds that feed on earthworms according to EFSA [16].

In addition to log K_OW_ and BCF, information on biomagnification in aquatic and terrestrial food chains can provide important information for assessing bioaccumulation potential of chemicals in many directives, regulations, guidelines or other official documents. In many directives, there are no legally binding threshold values for such data yet, but they can be used for qualitative assessment of bioaccumulation potential according to the amended Annex XIII of REACH [17]. In the future, criteria should be harmonised and improved in the different directives and regulations and field data should be more intensively used for assessing the bioaccumulation potential of chemicals.

### Use of monitoring data for bioaccumulation assessment

In addition to laboratory-derived BCF and BMF data, bioaccumulation can be assessed retrospectively by monitoring data if a chemical has already been in use and discharged into the environment. The implementation of the Stockholm Convention [18] particularly has promoted the use of monitoring data for bioaccumulation assessment. In this context, a range of substances were defined as bioaccumulative and are now listed as POP by the Stockholm Convention despite relatively low BCF values [19]. PFOS, for example, has only a moderate BCF of 240 to 1,300 but was assessed as bioaccumulative due to data on biomagnification in terrestrial and marine mammals. Monitoring data can be used for existing substances such as PFOS or HBCDD for which a ban is elaborated. For chemicals which have already been authorised (e.g. as biocides), knowledge on their concentrations in the environment can be used for potential reauthorisation or new authorisations (e.g. as veterinary medicine) in other legal frameworks (Bänsch-Baltruschat B, Claus E, Coors A, Duis K, Hommen U, Rüdel H, Keller K: Nutzung des Umweltmonitorings für das Risikomanagement bedenklicher Stoffe unter besonderer Berücksichtigung von PBT-Stoffen, submitted).

Several reviews discuss the application of monitoring-derived trophic biomagnification factors for characterising bioaccumulation potential of chemicals (e.g. [20,21]). Recently, Bänsch-Baltruschat et al. (submitted) have proposed a concept for assessing bioaccumulation potential of chemicals by using monitoring data. The authors report that in most cases, data on concentrations in the relevant matrix combinations, i.e. organism and surrounding medium, are not available. Either both matrices (e.g. fish and water) have not been measured in the same year or at the same station, or chemical concentrations in biota were not supplemented by measurements of the surrounding medium.

In the frame of the German Environmental Specimen Bank (ESB), samples of suspended particulate matter (SPM) and biota have been taken yearly and aliquot pool samples have been deep-frozen for retrospective monitoring since the 1980s [22,23]. The major advantage of the ESB is that material from different areas sampled over a period of up to 30 years is already available and can be used directly. BMF_field_ can, in principle, be determined for different matrix combinations: At fresh water stations, lipid-normalised chemical concentrations in the zebra mussel, *Dreissena polymorpha*, can be divided by organic carbon normalised chemical concentrations in SPM to obtain a biota/SPM accumulation factor for lower trophic levels. In the marine environment, organisms of different trophic levels are monitored by the ESB: the brown alga common bladder wrack (*Fucus vesiculosus*), the blue mussel (*Mytilus edulis*), eelpout (*Zoarces viviparus*) and egg content of the herring gull (*Larus argentatus*). For sites where a direct prey to predator relationship exists, BMF_field_ values can be calculated. Assuming that the difference between trophic levels in each prey to predator combination equals 1, fresh weight normalised biota concentration of PFOS resulted in a BMF_field_ of approximately 5 for herring gull/eelpout and 20 for eelpout/blue mussel (North Sea sampling site Jade bay/Mellum; based on data from [24]). Indeed, it has to be considered that in the different species either whole soft tissue or single tissues were investigated (e.g. whole soft tissue in mussels, liver in eelpout) which can vary in their bioaccumulation potential. Due to missing data, biota concentrations have also not been normalised to protein content, although PFOS is known to bind to proteins [25]. Furthermore, information on prey/predator trophic interaction and exact trophic positions of the studied species have to be confirmed (e.g. by stomach content analyses or carbon and nitrogen stable isotope ratio shifts (δ^13^C, δ^15^N); [20]).

### General approaches for analysing chemicals in biota

Today, the most common standard methodology to quantify the total content of trace metals and metalloids (metal(loid)s) in organisms is microwave-assisted acid digestion followed by inductively coupled plasma mass spectrometry (ICP-MS). However, for some analytes like mercury, direct methods for solids are available (e.g. cold vapour atomic absorption spectrometry method (CV-AAS)) [26]. Indeed, the sample preparation, acid digestion and dilution are critical factors in single organism or target organ analyses for most metal(loid)s if the organisms are very small. Coupling ICP-MS to an electrothermal vaporisation unit (ETV) may have the potential to be an alternative (direct) approach in total content multi-element analyses [27]. In the ETV, the sample is vaporised within seconds by heating in a graphite furnace up to approximately 2,000°C (this is comparable to CV-AAS or graphite furnace AAS). Advantages of the direct method are direct transfer of a dry aerosol to the plasma (no oxygen-based interferences from water), combined with multi-element capacity and high sensitivity of the ICP-MS. A lower effort for sample preparation is paid for with certain analytical drawbacks. Since it is still an analytical niche application, there is a lack of appropriate reference materials certified for homogeneity and concentration of various metal(loid)s.

Comparable to the sample preparation for metal(loid)s, the sample preparation for quantifying HOCs in biota is also very labour-intensive. After freeze-drying and extraction with organic solvents, clean-up of the extracts is performed to remove unwanted cell debris and tissue components that can interfere with the analytical measurements. Depending on the target analytes, the cleaned extracts are then analysed by, e.g. liquid chromatography or gas chromatography that are coupled to (quadrupole) mass spectrometry detection (LC-MS/MS or GC-MS/MS) (e.g. [28–30]). To improve the sensitivity of the method, small organisms are often pooled for one sample. Since HOCs are usually related to lipid content, this parameter should also be determined in the sample [31]. In both inorganic and organic analyses, the results are often transferred from one data processing level to another (e.g. from analyses to modelling) without transferring the uncertainties given at the respective precursor level. This often results in ‘seeming’ accuracies and reliabilities.

Regarding the new priority substances that have to be measured in biota for compliance checking with the WFD, European member states are presently elaborating guidelines for monitoring new EQS_biota_. Analysis of heptachlor/epoxide, HBCDD [32], PFOS [24] and PBDE [33] in aquatic biota has already been described.

### Monitoring for compliance checking with regulatory directives

Environmental contaminants are monitored in aquatic organisms, particularly in fish, for compliance checking with regulatory directives. In Europe, next to EQS_biota_, there are threshold levels for fish, fishery products and seafood according to European food laws such as the European Contaminant Regulation [34] and the recommendation 2006/88/EC of the European Commission [35]. Furthermore, national directives such as the German regulation on tolerable levels of contaminants in food [36] also define maximum allowable concentrations in food.

Monitoring chemicals in aquatic biota for food surveillance purposes differs from environmental monitoring: In food surveillance, the subject of protection is solely the human being as consumer. Usually fish, fishery products or seafood are obtained from commercial markets so that the allocation to the ecosystem often remains unclear. In general, chemicals are measured in muscle tissue and refer to fresh weight of the sample. For polychlorinated dibenzodioxins, polychlorinated dibenzofurans and dioxin-like PCBs, values are indicated in toxic equivalents according to the World Health Organisation (WHO-TEQ). Depending on the consumption pattern, analysing muscle tissue with skin may even be appropriate. In the European water framework directive, however, the subjects of protection are either humans or predators [37]. The latter mainly feed on the whole body of smaller fish. If comparing data from food surveillance and environmental monitoring, such aspects need to be considered.

In Europe, member states have to measure chemicals in aquatic biota for compliance checking with EQS_biota_ [2,3]. The guidance document for chemical monitoring of sediment and biota [37] gives recommendations for implementing this monitoring. The German Working Group on water issues of the Federal States and the Federal Government (LAWA) has further elaborated a concept for monitoring EQS according to directive 2008/105/EG and gives recommendations, e.g. for selection of fish species and size of sampled fish [38]. In fish, contaminant concentrations are usually measured in muscle tissue and refer to the fresh weight of the sample. Since the common eel (*Anguilla anguilla*) accumulates high amounts of chemicals due to its food habits and its high lipid content, monitoring data are often presented separately for this species. In the Rhine River, for example, next to the common eel, the white fish species roach (*Rutilus rutilus*), common bream (*Abramis brama*) and chub (*Squalius cephalus*) are often monitored [39]. In fish, levels of mercury and mercury compounds usually exceed the European EQS_biota_ of 20 μg kg^−1^ fresh weight whereby recent pollution is mainly ascribed to the diffuse atmospheric input [33,40]. In Bavaria, for example, 98% of the fish muscle samples exceeded the EQS_biota_ for mercury in 2007 to 2009 [41]. For trend monitoring, European member states further have to analyse 14 priority substances such as hydrophobic organic chemicals, metals and tributyltin in sediment, suspended particulate matter and/or biota that tend to accumulate in these matrices [2]. In this context, sampling the same species as well as comparable size and age of fish are particularly important since these parameters affect bioaccumulation and may confound or even superimpose temporal trends. For chemicals that show increased levels in liver compared with muscle tissue of fish, analysing liver tissue may be advantageous.

### Investigative monitoring

In contrast to compliance monitoring with fish, an investigative monitoring with zebra mussels (*Dreissena polymorpha*) in Bavaria (Germany) has shown that mercury concentrations in this species is generally below EQS_biota_. In the frame of this monitoring program, zebra mussels were transposed from relatively unpolluted waters to a number of Bavarian rivers and lakes for 6 months. Mussels can hint at more local pollution events, e.g. upstream or downstream of a discharge, while fishes integrate the pollutant load over their whole migration area. Only at one site, the EQS_biota_ for mercury was exceeded in zebra mussels in 2007 to 2011 indicating a very local input of mercury [41].

Another example for the identification of chemical sources is monitoring triphenyltin (TPT) in Lower Saxony (Germany): After finding high tributyltin (TBT) and TPT concentrations in sediments and biota from a marina, concentrations of both chemicals were measured in fish at around 100 stations in Lower Saxony (Germany) in 1996. TPT that has been used until the mid-1990s as component in antifouling paints as well as fungicide showed higher levels in fish livers than TBT. Since the highest TPT levels were found in the livers of roach sampled in the Lüneburg Heath, Germany's largest production area for potatoes, this indicates a contamination with TPT due to its application as a fungicide [42].

### Event-related monitoring

Monitoring bioaccumulation can also support risk assessment of various natural or anthropogenic events such as floods or dredging activities. For the maintenance of waterways, dredging of sediments may be necessary. As an example, the amount of sediments had increased to an unacceptable level in the Hamburg Port in 2004. In consequence, 6.5 Mio. m^3^ of sediments were dredged from the lower Elbe river in the Hamburg Port area and disposed 25 km north-west of the island Scharhörn in the inner German Bight between 2005 and 2010. To assess the potential ecological impacts on the marine environment, a comprehensive monitoring program has been established [43]. Among many other parameters, bioaccumulation of metals and organic chemicals has been measured in two benthic invertebrates (mussels (white furrow shell, *Abra alba*) and snails (common whelk, *Buccinum undatum*)) and fish (dab, *Limanda limanda*) sampled in and around the disposal site as well as in reference sites. These species were selected since they are abundant in the monitoring area and they represent different trophic levels. Since 2008, significantly increased levels of the organotin compounds monobutyltin (MBT) and dibutyltin (DBT) were detected in snails from the disposal site hinting at an increased bioavailability of contaminants due to the sediment disposals. Indeed, bioaccumulation decreased again after cessation of the disposals in 2010: concentrations of the DDT metabolites p,p′-DDD and p,p′-DDE that had increased in snails sampled at the disposal site attained the same level as in reference sites in 2011. Sediment disposals had, in contrast to snails, no clear effect on concentrations of chemicals in fish and mussels. This shows that the selection of monitoring species can influence the outcome of monitoring studies.

### Passive sampling

Species-specific differences in bioaccumulation potential also have to be considered when the same species is not available in the whole monitoring area, such as the different river basins within Europe. In addition to species, bioaccumulation depends on further biotic (e.g. size, age, sex and physiological conditions of organisms) as well as abiotic parameters that have to be taken into account when evaluating chemical concentrations in biota. Passive sampling suffers less from the variability connected to biota and may in some cases complement biota monitoring. Passive sampling devices have a sampling phase, usually a polymer that accumulates chemicals when exposed in the environment or to an environmental sample. By quantifying concentrations of target analytes in the polymer of the sampler, freely dissolved concentrations (*c*_free_) are determined that are a measure of contaminant bioavailability. Due to the enrichment of target analytes within the passive sampling polymer, quantification limits of passive sampling techniques are often lower than those for conventional techniques (e.g. quantification of HOCs in water) [44–46].

Passive sampling in the water phase is generally conducted in the kinetic uptake regime, and time averaged concentrations can then be determined by *in situ* calibration with performance reference compounds [47,48]. These are dosed to the sampling phase prior to exposure, and their release in water is measured to calculate uptake rates of target compounds [49]. Since 2001, monitoring by passive sampling in water has been applied in parallel to a mussel watch program in the coastal area of the Netherlands. Mussels and samplers are deployed for 6 weeks in autumn and winter periods. *c*_free_ of PCBs derived from passive sampling show a strong relation with lipid-normalised concentrations in mussels (*Mytilus edulis*) [50].

A non-target application of passive samplers on laboratory scale is the investigation of waste water for the detection of potentially bioaccumulative substances according to OSPAR's whole effluent assessment concept [51]. In this context, a small polymer-based sampler is exposed to the wastewater sample for 24 h [52,53] Paschke et al. in [1]. All accumulated substances are quantified by gas chromatography, summarised and normalised to the reference compound 2,3-dimethylnaphthalene (log K_OW_ = 4.4).

In sediments, *c*_free_ of HOCs can be quantified by equilibrium sampling devices [54]: The polymer of the sampling device is brought in contact with the sediment until equilibrium of the target analytes between sediment and polymer is attained. Provided that the polymer does not deplete the analyte concentration in the sediment, analyte concentrations in polymer (*c*_polymer_) can be translated to freely dissolved concentration (*c*_free_) in sediment interstitial water:

7 $$ {c}_{\mathrm{free}}=\frac{c_{\mathrm{polymer}}}{K_{\mathrm{polymer},\mathrm{water}}} $$

When applying equilibrium sampling of HOCs in sediments, equilibrium partitioning concentrations in lipids (*c*_lipid ⇌ sediment_) can even directly be calculated by *c*_polymer_ and a compound-specific lipid to polymer partition coefficient (*K*_lipid,polymer_):

8 $$ {c}_{\mathrm{lipid}\kern0.24em \rightleftharpoons \kern0.24em \mathrm{sediment}}={c}_{polymer}\times {K}_{lipid, polymer} $$

Recent field studies have shown that actually measured lipid-normalised concentrations in fish were near or below these equilibrium partitioning concentrations in lipids [55,56], Schäfer S, Antoni C, Möhlenkamp C, Claus E, Reifferscheid G, Heininger P, Mayer P: Equilibrium sampling of polychlorinated biphenyls in river Elbe sediments – linking bioaccumulation to sediment contamination, submitted]. Partitioning coefficients, *K*_polymer,water_ and *K*_lipid,polymer_, that are essential for passive sampling and derivation of, e.g. *c*_free_ and *c*_lipid,partitioning_ are determined in laboratory experiments. Indeed, for emerging chemicals as well as for many polymers that are applied for passive sampling, appropriate partition coefficients are often not yet available. Nonetheless, direct measurements of *c*_free_ with equilibrium sampling techniques have the potential to substantially improve risk assessment and management of sediments contaminated with HOCs [57].

There are also various types of passive sampling devices for metal(loid)s with diffusive gradients in thin films (DGT) [58,59], dialysis based techniques [60] and Chemcatcher® [61,62] being most commonly applied. DGTs are kinetic regime sampling devices that consist of a thin hydrogel layer that constrains diffusive transport of solutes into a binding layer [58]. The chemical mass absorbed by the binding layer in a given time is used for calculating *c*_free_ in water, sediment or soil. DGT devices can also be applied for assessing the long-term release and bioavailable fraction of metal(loid)s from construction products in hydraulic engineering (e.g. in waterways). In contrast to standardised batch experiments that require the repeated renewal of the water phase, in DGT-based long-term batch experiments, the amount of ionic metal(loid) species is continuously reduced to avoid equilibrium in the test medium which impacts the further release from the test material [63].

## Conclusions

The significance of bioaccumulation for assessing the chemical status of bodies of water is increasingly acknowledged as exemplified by setting new EQS_biota_ in the amended WFD EQS directive. Implementing the monitoring for compliance checking with these new EQS_biota_ is one of the challenges for the EU member states in the near future. As previously stated by Fuerhacker [64], the EQS directive is valuable for approaching a good chemical status of bodies of water even though new chemicals are not adequately considered. We further suggest that for bioaccumulative and non-metabolisable substances, EQS_biota_ are more relevant to chemical water quality than EQS_water_ since internal chemical concentrations are more related to a chemical's uptake and toxicity. Additionally, biota monitoring results can be related to ecological water quality as demonstrated by Van Ael et al. [65]. The authors showed that for most investigated chemicals, ecological water quality being assessed by fish community structure was lower when chemical concentrations in fish were elevated. However, due to limited resources, the European member states have implemented biota monitoring for compliance checking with the WFD on a smaller scale than monitoring of the water phase and sediment.

Next to compliance checking with regulatory directives, chemical concentrations in biota can be used for identifying sources of contamination and event-related monitoring. Since bioaccumulation of chemicals is closely linked with their toxicity, monitoring bioaccumulation can improve risk assessment of environmental contamination.

When monitoring chemical concentrations in biota, various abiotic and biotic factors that can affect bioaccumulation have to be considered. Since monitoring programs have different conceptual and methodological approaches, these have to be taken into account when comparing data from different programs. Assessing bioaccumulation of hydrophobic organic chemicals can be improved by additionally measuring lipid content of the analysed tissue. We further suggest to elaborate consensual standardised protocols for chemical analyses of biota and to perform quality assurance by inter-laboratory exercises.

We have highlighted that passive sampling can complement biota monitoring that aims at protecting human health and predators. It can also be helpful in spatial and temporal trend monitoring of chemical status of bodies of waters since samplers with standardised partition properties can be used over a wide geographical and temporal range.

Strategies for assessing bioaccumulation potential of chemicals need to be further optimised and harmonised for different regulations and groups of chemicals. *In vitro* tests are needed for preliminary testing of bioaccumulation potential. Monitoring data can in principle be used for bioaccumulation assessment of chemicals, but monitoring programs have to be improved to deliver all necessary data.

Further, communication between monitoring and risk assessment communities needs to be improved to create awareness for critical issues and to mutually benefit from technical expertise and scientific findings. Scientific support is necessary for establishing new monitoring programs for bioaccumulation.

## Endnotes

^a^persistent, bioaccumulative and toxic substances (PBT).

^b^very persistent and very bioaccumulative substances (vPvB).

## Abbreviations

α: chemical assimilation efficiency
AAS: atomic absorption spectrometry
BAF: bioaccumulation factor
BCF: bioconcentration factor
BCF_SS_: bioconcentration factor at steady state
BMF: biomagnification factor
BMF_k_: kinetic biomagnification factor
BMF_field_: biomagnification factor derived from field data
*c*_aq_: concentration in water
*c*_free_: freely dissolved concentration
*c*_organism_: concentration in organism
*c*_polymer_: concentration in polymer
*c*_predator_: concentration in predator
*c*_food_: concentration in food
*c*_lipid,partitioning_: concentrations in model lipids at thermodynamic equilibrium with the sediment
CV-AAS: cold vapour atomic absorption spectrometry
EQS: environmental quality standard
EQS_biota_: environmental quality standard for biota
EQS_water_: environmental quality standard for water
ESB: environmental specimen bank
ETV: electrothermal vaporisation unit
GC-MS/MS: gas chromatograph coupled to (triple quadrupole) mass spectrometer
HBCDD: hexabromocyclododecane
HOC: hydrophobic organic chemical
Ι: feeding rate constant
ICP-MS: inductively coupled plasma mass spectrometry
k_2_: depuration rate constant
K_polymer,water_: polymer to water partitioning coefficient
K_lipid,polymer_: lipid to polymer partitioning coefficient
LAWA: German Working Group on water issues of the Federal States and the Federal Government
LC-MS/MS: liquid chromatograph coupled to (triple quadrupole) mass spectrometer
Log K_OW_: logarithmised 1-octanol to water partitioning coefficient
NOAEL_oral_: no observed adverse effect level after oral uptake
NOEC_oral_: no observed effect concentration for the food
PFOS: perfluorooctanesulfonic acid
POP: persistent organic pollutant
QS_biota_: quality standards for biota
QS_biota,secpois_: quality standard for biota derived for predator eating contaminated prey
QS_biota,hhfood_: quality standard for biota derived for humans eating fishery products
TBT: tributyltin
TPT: Triphenyltin
WFD: Water Framework Directive
